# Understanding the Impact of Polyunsaturated Fatty Acids on Age-Related Macular Degeneration: A Review

**DOI:** 10.3390/ijms25074099

**Published:** 2024-04-07

**Authors:** Maëlis Brito, Capucine Sorbier, Nathalie Mignet, Vincent Boudy, Gerrit Borchard, Gaëlle Vacher

**Affiliations:** 1 Unither Développement Bordeaux, Avenue Toussaint Catros, 33185 Le Haillan, France; 2 Université Paris Cité, CNRS, INSERM, UTCBS, Unité de Technologies Chimiques et Biologiques pour la Santé, F-75006 Paris, France; 3 Département de Recherche et Développement (DRDP), Agence Générale des Equipements et Produits de Santé (AGEPS), Assistance Publique Hôpitaux de Paris (AP-HP), 7 Rue du Fer-à-Moulin, 75005 Paris, France; 4 Institute of Pharmaceutical Sciences of Western Switzerland (ISPSO), School of Pharmaceutical Sciences, University of Geneva, Rue Michel-Servet 1, 1206 Geneva, Switzerland

**Keywords:** retina, photoreceptors, age-related macular degeneration (AMD), polyunsaturated fatty acids (PUFA), omega-3 fatty acids, AMD in vitro models, AMD in vivo models

## Abstract

Age-related Macular Degeneration (AMD) is a multifactorial ocular pathology that destroys the photoreceptors of the macula. Two forms are distinguished, dry and wet AMD, with different pathophysiological mechanisms. Although treatments were shown to be effective in wet AMD, they remain a heavy burden for patients and caregivers, resulting in a lack of patient compliance. For dry AMD, no real effective treatment is available in Europe. It is, therefore, essential to look for new approaches. Recently, the use of long-chain and very long-chain polyunsaturated fatty acids was identified as an interesting new therapeutic alternative. Indeed, the levels of these fatty acids, core components of photoreceptors, are significantly decreased in AMD patients. To better understand this pathology and to evaluate the efficacy of various molecules, in vitro and in vivo models reproducing the mechanisms of both types of AMD were developed. This article reviews the anatomy and the physiological aging of the retina and summarizes the clinical aspects, pathophysiological mechanisms of AMD and potential treatment strategies. In vitro and in vivo models of AMD are also presented. Finally, this manuscript focuses on the application of omega-3 fatty acids for the prevention and treatment of both types of AMD.

## 1. Introduction

Age-related Macular Degeneration (AMD) is a chronic retinal disease affecting people over 50 years old. Two forms are distinguished: the atrophic (so-called dry or non-exudative) form and the neovascular (so-called wet or exudative) form. Patients are subjected to the degradation of the photoreceptors of the macula, the central zone of the retina, causing a decrease in visual acuity. In the later stages, black spots occur in the center of the field of vision, resulting in “legal blindness” with a visual acuity below 20/200 [1]. AMD is one of the leading causes of blindness in developed countries and is responsible for 8.7% of new cases of blindness worldwide [2].

To this day, wet AMD is treated with anti-vascular endothelial growth factor (VEGF) drugs prescribed as first-line therapy, unlike dry AMD, for which there is no real effective treatment available in Europe. Moreover, these therapeutic strategies only allow to slow down the progression of the disease and are not curative treatments. Research is, therefore, underway to improve patient comfort in terms of effectiveness and route of administration thanks to new active pharmaceutical ingredients and novel formulations.

In recent years, fatty acids have been shown to have an impact on the pathophysiology of AMD and proved to be an interesting and effective therapeutic approach. Indeed, long-chain polyunsaturated fatty acids (LC-PUFA) and very long-chain polyunsaturated fatty acids (VLC-PUFA) are essential components of retinal photoreceptors, contributing to their proper development and function. In AMD patients, the quantity of these fatty acids is considerably reduced [3]. By understanding the mechanisms involved in this occurrence, supplementation in an appropriate formulation could be of major interest in both preventing and treating the disease.

## 2. Retina and Vision

### 2.1. Eye Anatomy and Principal Functionalities

**Figure 1:**
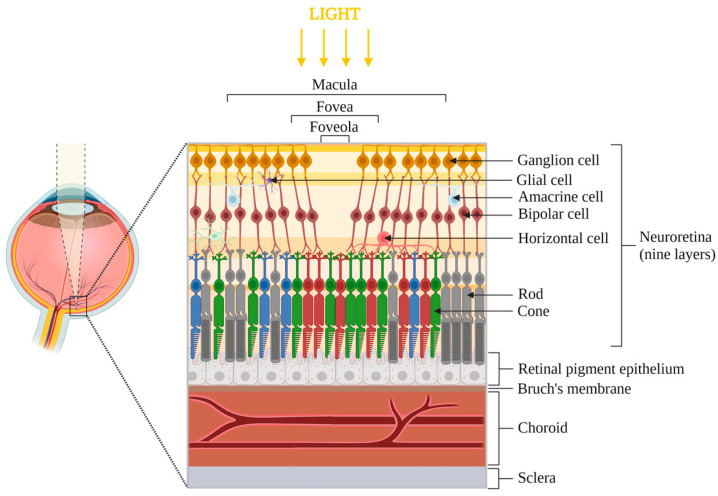
Anatomy of the posterior eye segment, focused on the central zone of the macula. The retina is divided into two parts: neuroretina (includes ganglion cells, amacrine cells, bipolar cells, horizontal cells, rods and cones photoreceptors and glial cells) and retinal pigment epithelium. Bruch’s membrane, choroid and sclera are located below the retina. The foveola is the most central zone of the macula, where the light directly reaches the photoreceptors. Note: elements are not to scale.

The eye is a sensitive and complex organ responsible for vision. It is, among other constituents, composed of three tunics (retinal, uveal, fibrous), two segments (anterior and posterior), a lens, and a cornea [4]. The retina is a thin transparent layer (<500 μm) that can be divided into two zones: the neuroretina and the retinal pigment epithelium (RPE) (Figure 1). The latter is attached to Bruch’s membrane, which serves as the inner wall of the choroid (300–500 μm) [5].

The macula represents the center of the neuroretina and is composed of numerous photoreceptors. It also contains macular pigments, lutein and zeaxanthin, that filter blue light and possess antioxidant properties [6].

Photoreceptors are polarized neuronal cells composed of phospholipids enriched with polyunsaturated fatty acids (PUFA) of variable length. Two types of photoreceptors can be distinguished, rods and cones, with an approximate physiological ratio of 20:1. Both types are divided into an internal segment, where the necessary material for metabolism is contained, and an external segment, in the form of stacks of discs for rods. External segments represent the photosensitive part containing photopigments derived from vitamin A [7,8].

Rods represent 95% of photoreceptors and enable vision in dim conditions (night). Approximately 5 million cones ensure high visual acuity and color vision in bright conditions (day) [9]. Blue, green and red cones ensure optimal light absorption at wavelengths of 430, 530 and 561 nm, respectively. Blue cones are less present in the retina and completely absent in the fovea. Rods and cones contain 11 cis-retinal, a photoreceptor chromophore, which is transformed into trans-retinal during the absorption of light photons. When excited by blue light, the retinal undergoes chemical transformation, resulting in toxic and oxidative metabolites for the eye [10,11].

The functions of the RPE include the transport of nutrients from the choriocapillaris to the photoreceptors, retinal waste management (phagocytosis of approximately 10% of the photoreceptor outer segment disks per day), photopigment recycling, participation in the metabolism of PUFA, vitamin A and derivatives, as well as the synthesis of growth factors such as VEGF [12,13].

Bruch’s membrane is a pentalaminar vessel wall located at the basal part of the RPE. It allows the bidirectional passage of nutrients, metabolites and waste products between the RPE and the choroid. Lipids and oxidized materials can accumulate in this area over time [14].

The choroid, located below Bruch’s membrane, contains numerous vessels, choriocapillaris, that allow the vascularization of the external areas of the retina [15], as well as the evacuation of photoreceptor waste phagocytosed by the RPE. The development of choriocapillaris is regulated by VEGF [16].

### 2.2. Physiological Aging

Like any part of the human body, the eye is affected by age, with cellular and structural changes. In addition, due to its low repair capacity, the slightest retinal damage can lead to serious degradation [17]. Natural aging leads to changes in the different areas of the retina: photoreceptors, RPE, Bruch’s membrane and choroid. This is clinically illustrated by a decrease in visual acuity, a reduced adaptation in dark environments as well as a decreased sensitivity to contrasts [18].

Some debris, called drusen, are characterized by small yellowish spots at the interface between the Bruch’s membrane and the RPE. Consisting of lipid granular extracellular material, they contain various compounds such as esterified and non-esterified cholesterol [19], serum amyloid P, amyloid β peptide, apolipoprotein E (ApoE), vitronectin, complement proteins and lipid peroxidation products [20,21,22]. They are not considered pathological as long as they are smaller than or close to 63 μm in size (hard drusen) [23]. Physiological ocular aging also leads to the appearance and accumulation of lipofuscins, characterized by intracellular lipid and protein aggregate wastes inefficiently digested by lysosomes [24]. Upon contact with light, lipofuscins, which are autofluorescent and photosensitive [25], are involved in the formation of reactive oxygen species (ROS). Indeed, bis-retinoid N-retinyl-N-retinylidene ethanolamine (A2E), a lipofuscin fluorophore, contributes to the apoptosis of RPE cells [12].

All these anatomical changes and the presence of inflammatory and oxidative phenomena result in an abnormal accumulation of metabolic and lipidic debris, excessive destruction of photoreceptors and the production of ROS, all of which are thought to be responsible for ocular pathologies such as AMD.

## 3. AMD: Clinical Aspects

Due to the excessive degeneration of photoreceptors (mainly rods) in the macula [26,27], AMD is characterized by a progressive loss of central vision in one or both eyes. It is estimated that 288 million people worldwide will suffer from AMD by 2040, even though the prevalence has been declining over the past 20 years due to improvements in lifestyle and healthcare [28].

### 3.1. Risk Factors

Several factors influence the risk of developing AMD, the most important one being age.

Genetic factors (family history, mutations) may also have a negative impact on the occurrence of the disease. Polymorphisms in genes coding for complement factor H (CFH) [29] or proteins of complement components 3 and 5 (C3, C5) (involved in inflammation) [30,31] are reported in AMD patients. ARMS2 and HTRA1 (linked to oxidative stress) have also been associated with AMD [32]. Apolipoprotein E, involved in lipid homeostasis and functioning as a lipid and cholesterol transporter [33], has three allelic variants, from the most frequent to the less frequent in the human body: ApoE3, ApoE4 and ApoE2 [21]. Studies show that among these polymorphisms, the ApoE2 allele increases the risk of developing subretinal neovascularization [34], while ApoE4 seems to be protective [35]. On the contrary, Liutkeviciene et al. suggested that ApoE4/E2 genotype could be protective [36], Viturino et al. found no impact of ApoE2/E2 genotype on the increased risk of AMD [37], and Fernández-Vega et al. described a protective role of ApoE2 allele in wet AMD in a Spanish population [38]. Thus, these ApoE polymorphisms and their specific roles in AMD should be further studied.

Environmental factors are involved in the appearance and progression of the disease. Smoking, which increases the risk of AMD by two to four times [39], is considered the first environmental risk factor in AMD [40]. On one hand, plasmatic levels of high-density lipoprotein (HDL) cholesterol are associated with the occurrence of AMD [40]. On the other hand, a Mediterranean diet decreases this risk; however, this is strongly dependent on the patient’s genotype [41,42]. The impact of light, mainly blue light, as a risk factor for AMD still remains controversial [43,44,45,46].

### 3.2. Evolution of AMD

Age-Related Maculopathy (ARM) corresponds to the early stage of the disease (early AMD), before the onset of macular degeneration. The retina does not present any hyper- or hypopigmentation [23], but drusen begins to show [47,48], ranging in size from 63 to 125 µm, with outlines harder to define (soft drusen) [23]. With the progression of ARM, the pathology may switch to intermediate AMD.

Intermediate AMD is characterized by an increase in the drusen size beyond 125 μm. Hyper- or hypopigmentation becomes visible [23].

**Figure 2:**
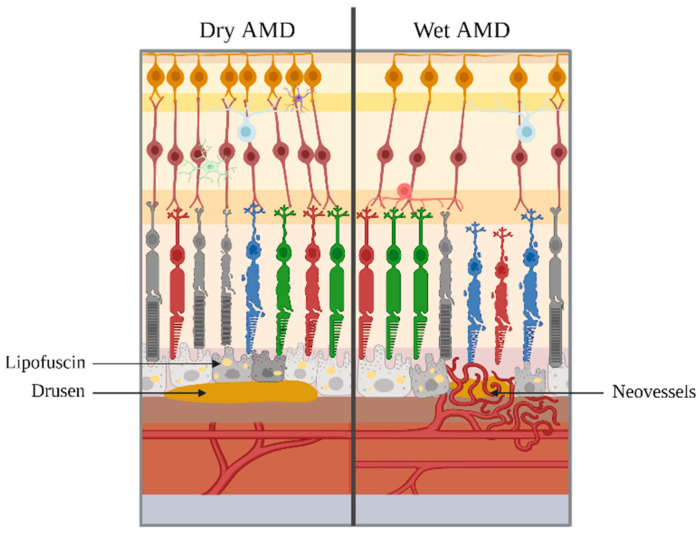
Final stages of AMD. On the left, dry AMD is represented, with the presence of lipofuscins and drusen. On the right, wet AMD is represented, with the supplementary appearance of neovessels. Note: elements are not to scale.

Late AMD can be distinguished into dry AMD and wet AMD (Figure 2). The dry-to-wet AMD ratio is estimated at 85:15 [49]. This pathology is complex; the dry form can evolve into a wet form and vice versa. Moreover, patients can also have a dry form in one eye and a wet form in the other.

Dry AMD, also known as nonexudative or atrophic form, is the most common form of AMD, which progresses slowly over time. It is typically characterized by the presence of soft drusen between the RPE and Bruch’s membrane, accompanied by pigmentary alterations, destruction and dysfunction of RPE cells. Disease progression, which is spread over an average time span of 5 to 10 years, corresponds to the degeneration and atrophy of the retina. This geographic atrophy (GA) is the result of the apoptosis of photoreceptors, leading to the loss of vision [48].

Wet AMD, also called exudative or neovascular form, is the most aggressive form and can lead to blindness within a few weeks or months if not treated. Wet AMD is characterized by the abnormal growth of neovessels originating from the choroid [50,51] that develop into Bruch’s membrane and can cross the RPE [52]. This leads to fluid leakage (e.g., serum and blood), resulting in retinal hemorrhages, RPE detachments or retinal serous detachments, resulting in photoreceptor degeneration and loss of vision [53]. This neovascularization may be associated with the presence of drusen and lipofuscins deposits. The appearance of subretinal fibrous scarring marks the final and irreversible progression of the neovascularization process [54].

## 4. AMD: Pathophysiology

Under the influence of various pathological mechanisms, including inflammation and oxidative stress [55,56], physiological ocular aging can switch to pathological ocular aging, responsible for AMD. Rozing et al. proposed the following mechanism: first, cellular damage, due in part to oxidative stress, could be responsible for a decrease in the function of the retina. This phenomenon would then activate an inflammatory process, further exacerbating the changes observed during natural aging and leading to pathological aging [27].

### 4.1. Oxidative Stress

The role of oxidative stress in the pathogenesis of AMD is confirmed by studies that show that smoke, a source of oxidative stress, is one of the main environmental risk factors for AMD [57]. Also, the absence of a redox-sensitive protein called DJ-1 results in a lack of response to oxidative stress and, therefore, retinal dysfunction [58]. Moreover, the RPE and photoreceptors undergo oxidative stress due to their sensitivity to short-wave photons and to an environment with high levels of oxygen [24], resulting in the production of ROS by mitochondria and in the abnormal development of lipofuscins [59]. Its fluorophore, A2E, is involved in retinal degeneration [25]. Indeed, it plays a role in the apoptosis of RPE cells when exposed to blue light, as well as in the activation of the complement system. Its accumulation in the RPE and its toxicity in the eye amplifies a detrimental inflammatory response in AMD [60,61]. The P2X7 purinergic receptor may also play a role in retinal oxidative stress linked with amyloid β peptide accumulation [62].

### 4.2. Inflammation

Drusogenesis is a complex process closely linked to local inflammation [59]. Indeed, drusen contain many proinflammatory factors, such as ApoE, IgG and complement factors [48].

TNFα, a pro-inflammatory factor, deregulates the OTX2 gene that controls many functions of the RPE and ensures its homeostasis [63]. As with oxidative stress, P2X7 reception is involved in the inflammatory process [62].

Numerous other receptors present in the retina are responsible for the secretion of cytokines by the RPE, amplifying its degradation in a vicious circle. For example, Toll-like receptors (TLRs) are involved in the production of cytokine pro-forms such as IL-1β [64]. The latter remains inactive until the NOD-like receptor P3 (NLRP3) inflammasome regulates the activity of caspase-1, which can activate interleukin [65,66]. The NLRP3 isoform is also activated by drusen, lipofuscin, the A2E fluorophore or amyloid β peptide [64].

### 4.3. Choroidal Neovascularization

Choroidal vessel growth is physiologically regulated by a balance between anti-angiogenic (pigment epithelium-derived factor—PEDF) and pro-angiogenic (VEGF) factors, both secreted by the RPE [67,68].

In wet AMD, this balance is disturbed, with VEGF predominant in response to local hypoxic conditions [69]. This hypoxia may be caused by the excessive presence of drusen between the RPE and Bruch’s membrane.

VEGF-A is responsible for pathological ocular angiogenesis and exists in different isoforms: A-121, A-165 (most common in the retina), A-189 and A-206. They act primarily on the VEGFR1 and VEGFR2 receptor tyrosine kinases, the latter being the most involved in ocular pathology [51].

The abnormal development of choroidal neovessels between the RPE and Bruch’s membrane may also be the result of increased inflammation. Pro-inflammatory mediators such as IL-1β, TNF-α and IFN-γ cause the RPE to produce cytokines and chemokines such as IL-6, IL-8, TNF-β and VEGF [70].

### 4.4. RPE and Bruch’s Membrane Degradation

All the phenomena mentioned above are closely interlinked, amplifying the degradation of the RPE and Bruch’s membrane in a vicious cycle.

As a result of these oxidative and inflammatory events, the RPE is less able to eliminate photoreceptor waste products, with an exacerbated accumulation of lipofuscins and drusen. As for Bruch’s membrane, excessive renewal of extracellular matrix components leads to a loss of elasticity and resistance, while insufficient renewal reduces the bidirectional passage of nutrients and waste products [71].

The accumulation of these products leads to cellular hypoxia (responsible for neovascularization) and further impedes the bidirectional passage of nutrients and waste products across Bruch’s membrane. Non-recycled and non-eliminated waste amplifies oxidative stress and inflammation, which, in turn, amplifies the degradation of the RPE and Bruch’s membrane, accumulating more waste that will ultimately not be eliminated.

## 5. Dry and Wet AMD Treatments

Current treatments are mainly targeted towards wet AMD, the most aggressive form, which, without diagnosis or treatment, can lead to legal blindness in a few weeks or months. The major drawback is that these treatments target the consequence (neovascularization) and not the cause. For the atrophic form, the course of treatment is mainly preventive (antioxidants, anti-inflammatories). Multiple reviews have already listed current treatments but also older ones, as well as molecules that were in development. This part of the review will, therefore, give a brief overview of the commercialized treatments in Europe and the molecules currently in development worldwide (Table 1).

## 6. Representative Models of AMD

### 6.1. In Vitro and Ex Vivo Models

To study the potential efficacy of a new molecule in the treatment of AMD, ocular cell-based models represent an efficient, reproducible and repeatable alternative to animal experiments. The cells can be primary or immortalized, the latter being used more frequently because of their low cost and their ability to multiply many times while maintaining their phenotype.

Cell lines can be subjected to different environments or stimuli, with the objective of reproducing and understanding the mechanisms involved in pathology. It then becomes possible to screen several drug formulations that would be of interest in the treatment of the disease. For AMD, the cells of interest are retinal and choroidal cells.

Cell-based models representing a pathological environment can be created through the activation or inactivation of a specific mechanism. For example, cell lines WI38 and IMR90, representative of aging cells, have been treated with a cytidine analog that inhibits DNA methyltransferase to study the expression of ELOVL2, an elongation enzyme involved in AMD [100]. A simple way to mimic wet AMD angiogenesis is the addition of VEGF to the cells, as studied by Wei et al., with human choroidal microvascular endothelial cells [101].

Primary pathological cells have the advantage of being collected directly from AMD patients. They allow a reliable reproducibility of the disease compared to immortalized cells. Voisin et al. compared hiPSC-RPE from dry AMD patients and elderly healthy individuals under normal conditions and oxidative stress. When these cells were subjected to oxidative stress by the application of ferric nitrilotriacetate, ROS production was higher for cells from AMD patients with higher cell degeneration [102]. In the presence of human serum of specific composition, healthy human fetal RPE cells develop subcellular deposits containing characteristic drusen molecules and complement proteins [103]. Gorham et al. used this model to evaluate the efficacy of a peptide in the inactivation of the complement system [104].

As AMD is a complex disease with numerous pathophysiological interactions between the retina and choroid, it has become of great interest to study more complex cellular systems such as co-cultures or organ-on-a-chip models.

De Cillà et al. evaluated the efficacy of aflibercept and ranibizumab under oxidative conditions on RPE cells (ARPE-19 cell line) co-cultured with human umbilical vascular endothelial cells (HUVEC) [105]. With primary cells, Palanisamy et al. co-cultured human primary RPE and human primary choroidal endothelial cells on Transwell inserts and concluded that this model could be useful for the screening of new molecules and permeability studies [106]. From this general model of co-culturing RPE and vascular endothelial cells, some authors replaced the culture insert with a microfluidic channel [107,108,109]. With this device, Chen et al. studied the co-culture of ARPE-19 and HUVEC cell lines and found that the secretion of VEGF was elevated under hypoxic conditions and the presence of a low-glucose medium [110]. Different authors developed retinal organoids from human induced pluripotent stem cells (iPSCs) through different protocols, with the final objective of studying the pathogenesis of the retina [111,112,113].

At the boundary between in vitro and in vivo tests, ex vivo experiments may be performed. Fietz et al. developed a model of a primary porcine RPE monolayer cocultured with porcine retinal organ cultures [114]. They found that this co-culture induced inflammation and could, therefore, be a reliable inflammatory AMD model. Labrador-Velandia et al. co-cultured porcine neuroretina and human mesenchymal stem cells to study neuroretinal degeneration [115].

### 6.2. In Vivo Models

As animal experiments are still essential for further evaluation of the efficacy and toxicity of a new treatment, in vivo models of dry or wet AMD are still being developed.

Mice are the most used species due to their low cost, rapid reproduction cycle and easily inducible genetic modifications, but the anatomy of their eyes does not allow for good predictability and transposition to human eyes [116].

Rabbits are, therefore, more suitable for pharmacokinetic studies. Indeed, their eyes are more similar to those of humans, with a corneal thickness of 375–385 μm [117] (compared to around 535 μm in humans [118]), an anterior chamber volume of 280 μL [119] (compared to approximately 130–170 μL [120]) and a vitreous volume of 1200–1500 μL [121] (compared to 4–5 mL [122]). The retinal thickness is around 120–160 µm in rabbits [123] and approximately 260 µm in humans [124].

However, there are still important differences, the main one being the absence of macula in numerous animals [125], which is crucial in the pathogenesis of AMD in humans. As animal models of AMD are, therefore, based only on pathological similarities, it complicates the correlation and interpretation of pharmacokinetics or efficacy of molecules between animal models and humans.

Transgenic mice with an ELOVL2 C234W mutation that deregulates the activity of the elongation enzyme ELOVL2 present sub-RPE lipid deposits characteristic of dry AMD [100].

Other models focus on the complement system, such as CFH-deficient (CFH^-/-^) mice [126], overexpressing C3 [127] or expressing a specific CFH variant, Y402H [128].

Animal models could also mimic oxidative stress, which is involved in the pathogenesis of AMD. For example, the carboxyethylpyrrole molecule is responsible for oxidative stress. This adduct is present in abnormally high quantities in AMD patients, following the oxidation of a fatty acid present in the photoreceptors, docosahexaenoic acid (DHA) [129]. The nuclear factor (erythroid-derived 2)-like 2 (NRF2^-/-^) mice model is responsible for the increase in carboxyethylpyrrole [130]. Tobalem et al. immunized mice with murine serum albumin conjugated with carboxyethylpyrrole. After three months, the treated mice presented signs of RPE degeneration. According to the authors, this model could mimic the natural progression of dry AMD in humans [129].

Mice with the ApoE4 allele on a high-fat diet develop the characteristics of AMD: drusen-like deposits, Bruch’s membrane thickening, RPE degeneration and, in some cases, choroidal vascularization. This ApoE isoform seems to accelerate the accumulation of the amyloid protein [131]. In rabbits, a cholesterol-enriched diet results in increased levels of ROS, amyloid β, apparition of drusen-like debris and cholesterol accumulation in the retina [132].

Mice deficient in the C-C motif chemokine ligand 2 cytokines and C-X3-C motif chemokine receptor 1 (CCl2^-/-^/CX3CR1^-/-^) [133], in CCl2^-/-^ alone or C-C chemokine receptor type 2 (CCR2^-/-^) receptors [134], show similar morphological, functional and structural characteristics of dry AMD. However, these similarities should be interpreted carefully. Indeed, Luhmann et al. found that the “drusen-like” deposits described in previous CCl2^-/-^ models [134] seem to be lipofuscin-containing macrophages. Moreover, these same authors found that the susceptibility of developing CNV is reduced in this model [135].

Yasukawa et al. developed a rabbit model to study the biogenesis of drusen. They mimicked the accumulation of lipofuscin by injecting subretinally glycoxidized microspheres and hypothesized that lipofuscin could contribute to the appearance of drusen. They observed that rabbits injected with these microspheres developed drusen-like deposits more frequently [136].

To study wet AMD, subretinal injections of pro-angiogenic factors could be of interest [137], as well as laser-induced CNV thanks to the application of a laser in the retina and choroid of mice, rats or rabbits [138,139,140,141].

Studies on amphibians were carried out to reproduce AMD by mechanically suppressing outer segment photoreceptors or by locally injecting an antibiotic that destroys cones and rods [142].

Thus, rodent models are of great interest to study genetic and metabolic alterations, mostly in dry AMD. Rabbits are useful for screening different molecules and treatments against dry and wet AMD [125]. However, none of these models allow the development of a complete and reliable model of human AMD. Research must, therefore, continue in this field.

## 7. The Specific Role of Fatty Acids in AMD

### 7.1. Fatty Acids in Humans

Fatty acids are molecules consisting of a hydrocarbon chain of variable length with a terminal carboxyl function. They are essential to the human body and have numerous functions, such as providing energy (through storage in the form of triglycerides), constituting cell membranes or improving cardiovascular health [143,144].

Fatty acids can be found in different forms in the body: free fatty acids, bound to triglycerides or phospholipids and included in lipoproteins. Inactive in the bloodstream, fatty acids exert an action in tissues in which they are incorporated [145].

They are differentiated by their chain length and the presence of double bonds. Short-chain fatty acids (between 2 and 4 carbons), medium-chain fatty acids (between 6 and 12 carbons), long-chain fatty acids (LC-FA, between 14 and 24 carbons) and very long-chain fatty acids (VLC-FA, ≥26 carbons) can, therefore, be distinguished. The presence or absence of double bonds makes it possible to differentiate saturated fatty acids (SFA) with no double bonds from monounsaturated fatty acids (MUFA) with a single double bond and polyunsaturated fatty acids (PUFA) with at least two double bonds. Moreover, the position of the first double bond subdivides PUFA into omega-3 (first double bond on the third carbon from the methyl group) and omega-6 (first double bond on the sixth carbon from the terminal methyl group). MUFA are called omega-9 when the first double bond is on the ninth carbon from the terminal methyl group.

**Figure 3:**
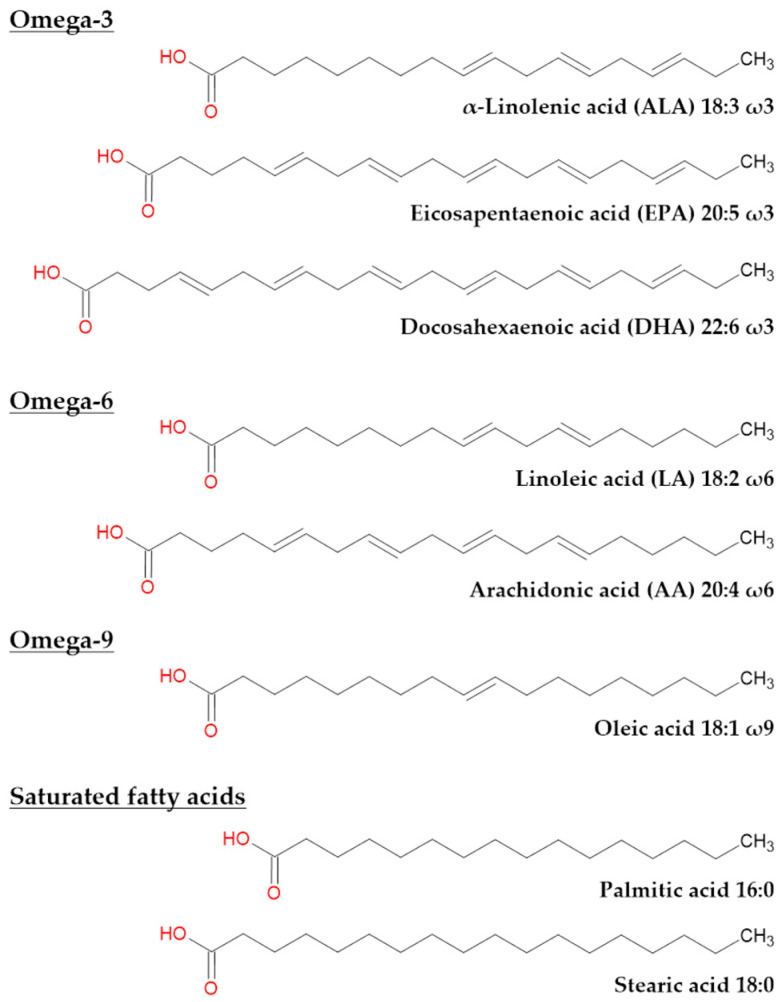
Chemical representation of most common fatty acids found in the eye and their precursors. ALA and LA are precursors of omega-3 and omega-6 PUFA, respectively. EPA, DHA, AA, oleic acid, palmitic acid and stearic acid are common fatty acids in the eye.

Essential fatty acids are LC-PUFA, which the body is not able to synthesize de novo and must be supplemented by diet. Indeed, humans do not possess the Δ15 and Δ12 desaturases [146] necessary for the synthesis of essential fatty acids from oleic acid 18:1 ω9. This includes linoleic acid (LA) 18:2 ω6 and α-linolenic acid (ALA) 18:3 ω3 (Figure 3). LA, the precursor of all omega-6 LC-PUFA, is found in some vegetable oils such as soybean oil but also in sunflower seeds, Brazil nuts and eggs [147]. ALA, the precursor of all omega-3 LC-PUFA [148], can be found in other vegetable oils such as flaxseed oils, but also chia seeds and quinoa [149].

In addition, some LC-PUFA, other than essential fatty acids, can be incorporated into human diet. The main plant source of omega-3 LC-PUFA is algae that fish feed on. For humans, omega-3 fatty acids are, therefore, found in fatty fish [143]. Omega-6 fatty acids are found in red meat and oils such as sunflower or palm oil.

In the eye, five LC-FA are the main constituents of the retina: palmitic acid 16:0, stearic acid 18:0, oleic acid 18:1 ω9, arachidonic acid (AA) 20:4 ω6 and DHA 22:6 ω3 [3] (Figure 3), the latter being the most present. Also, eicosapentaenoic acid (EPA) 20:5 ω3 plays a role in the fatty acid metabolism and retinal function. In total, unsaturated fatty acids account for approximately half of all retinal fatty acids [150].

### 7.2. LC-PUFA and VLC-PUFA Metabolism

In humans, LC-PUFA and VLC-PUFA are mainly but not exclusively found in the retina, brain, skin and testes [151]. LC-PUFA, such as EPA and DHA, can be produced by the human body, the main site of synthesis being the liver [152]. Local retinal synthesis exists but is slow and insufficient to ensure all needs of LC-PUFA in the retina [153]. VLC-PUFA are not present in the diet of vertebrates and must, therefore, be synthesized locally because the liver does not have the required enzymes [154]. In the retina, VLC-PUFA can be produced thanks to the elongation enzyme expressed in the inner segment of cones and rod photoreceptors [155].

**Figure 4:**
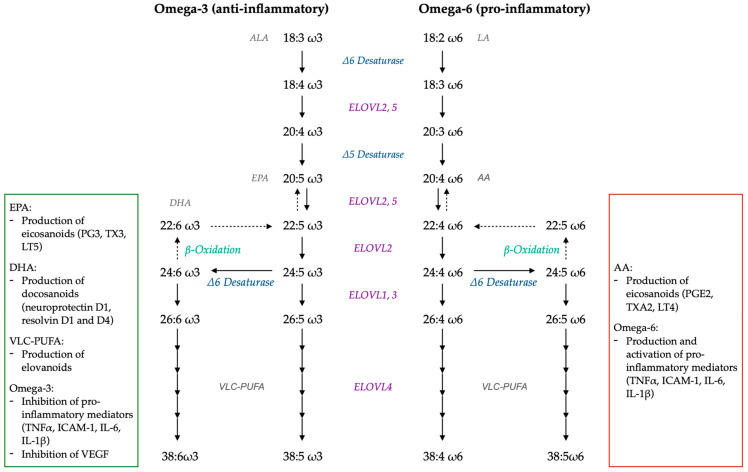
Metabolic pathway to produce PUFA (ω3 and ω6); roles of pro- and anti-inflammatory fatty acids. From ALA and LA, ELOVL2 and 5, Δ5 and Δ6 desaturases produce LC-PUFA. From LC-PUFA, ELOVL1, 3 and 4 produce VLC-PUFA. β-oxidation can occur and lead to the production of DHA or its ω6 equivalent. ω3 PUFA are mostly anti-inflammatory, while ω6 PUFA are mostly pro-inflammatory. Modified from [161].

Figure 4 represents the physiological metabolic pathway of PUFA in the retina. More precisely, essential fatty acids (ALA and LA) are elongated by Δ5 and Δ6 desaturases, ELOVL2 and ELOVL5. The ELOVL enzymes, comprising seven isozymes, condense an acyl-coenzyme A or acyl-CoA, and a malonyl-CoA into a 3-ketoacyl-CoA, which is then reduced and dehydrated. This elongation allows the production of a fatty acid with two additional carbons [156]. The elongation of fatty acids takes place in four steps: (i) condensation: this is a rate-limiting step, with the formation of 3-ketoacyl-CoA using ELOVL enzymes; (ii) reduction: conversion of 3-ketoacyl-CoA into 3-hydroxyacyl-CoA with nicotinamide adenine dinucleotide phosphate (NADPH) as a cofactor; (iii) dehydration: conversion of 3-hydroxyacyl-CoA into trans-2-enoyl-CoA thanks to 3-hydroxyacyl-CoA dehydratase; and (iv) reduction: trans-2-enoyl-CoA is converted into acyl-CoA, thanks to a reductase and NADPH as a cofactor [156]. PUFA obtained by these elongations are omega-3 (e.g., EPA, DHA) and omega-6 (e.g., AA) LC-PUFA, between 20 and 24 carbons. Once LC-PUFA is produced, ELOVL1, 3 and 4 elongate them to create VLC-PUFA between 26 and 38 carbons [154]. Among the different LC-PUFA, EPA is preferentially used as a substrate for ELOVL4, followed by AA and DHA [157].

ELOVL2 is a promising biomarker for aging [158] and is involved in AMD. When ELOVL5 is deleted in mice, there is a reduction of DHA and AA [159]. Mutations in ELOVL4 lead to a reduction of the VLC-PUFA amount in the retina and are at the origin of ocular pathology in young adults [160].

### 7.3. Retinal LC-PUFA and VLC-PUFA: Physiological Role

The retina is rich in LC-PUFA and VLC-PUFA, which are mainly present in the discs of photoreceptor outer segments [162].

**Figure 5:**
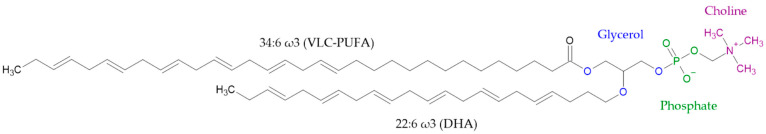
Representation of a phosphatidylcholine in photoreceptors. VLC-PUFA is in sn-1 position and DHA is in sn-2 position.

PUFA are bound to phospholipids, mainly phosphatidylcholine, which represents 40–50% of retinal phospholipids. Usually, a VLC-PUFA is in the sn-1 position and a DHA is in the sn-2 position of the glycerol backbone of phosphatidylcholine [154,156] (Figure 5). Other retinal phospholipids are phosphatidylethanolamine (30–35%), phosphatidylserine (5–10%) and phosphatidylinositol (3–6%) [152]. PUFA can also be esterified as diacylglycerols or triacylglycerols [163].

Physiologically, the distribution of LC and VLC-PUFA differs between cones and rods but also between the center and the periphery of the retina [164]. In rod-dominant animals, the amount of LC and VLC-PUFA is higher than in cone-dominant animals with double the concentration of DHA, suggesting different mechanisms and lipid requirements between these two types of photoreceptors. In humans, for whom the retina contains both rods and cones, DHA and EPA represent, respectively, about 50% and less than 0.29% of fatty acids found in the outer segment disc membrane [3,165]. The low concentration of EPA in ocular tissues is explained by its rapid use to produce DHA, VLC-PUFA or eicosanoids.

The macula, enriched in cones, has less DHA than the periphery [166], and LC-PUFA and VLC-PUFA are more present in the disc outer segments than in the whole retina [167].

These fatty acids have an indispensable role in the composition and proper functioning of photoreceptors, allowing their physiological development.

For example, DHA is metabolized and integrated as a structural compound of photoreceptors, allowing good membrane fluidity and promoting conformational changes and regeneration of rhodopsin during phototransduction [152]. Also, by increasing the enzymatic activity of the lysosomal lipase of the RPE, DHA allows the hydrolysis of lipid waste, thus avoiding its accumulation in Bruch’s membrane [6]. Through the production of neuroprotectin D1, a DHA-derived mediator, photoreceptors survive longer in vitro [168]. This DHA derivative also protects the retina against RPE oxidative stress [169].

EPA and DHA, through the production of mediators, have an anti-angiogenic activity and limit the development of neovessels [170]. The antiproliferative activity of DHA decreases in the presence of antioxidants and increases in the presence of pro-oxidant agents, which indicates that DHA while possessing an antioxidant activity by itself in free form, needs to be oxidized to exert its antiproliferative activity [171].

Omega-3 LC-PUFA, mainly docosapentaenoic acid, promotes the accumulation of lutein and zeaxanthin that filter blue light and have antioxidant and anti-inflammatory properties [172].

VLC-PUFA are present in the neuronal synaptic vesicles of photoreceptors and play an important role in their membrane curvature, fluidity and integrity [173].

### 7.4. Retinal LC-PUFA and VLC-PUFA: Role in AMD

Liu et al. compared the retinas of AMD donors to non-AMD patients of the same age group and found that the concentrations of LC-PUFA and VLC-PUFA were significantly decreased in AMD patients [3]. This lack of fatty acids could be caused by three factors.

The first is related to dietary changes; essential fatty acids or LC-PUFA are no longer supplied to the body via the diet.

The second factor concerns a deficiency in the elongation enzymes ELOVL. Mainly ELOVL2 and ELOVL4 are involved in fatty acid elongation, but the activity of ELOVL2 decreases with age [100]. Consequently, if this enzyme is no longer or less functional, there will be reduced production of LC-PUFA and, therefore, less production of VLC-PUFA.

The last factor may be an early degradation of LC-PUFA and VLC-PUFA, partly due to a damaging environment caused by oxidative stress and reactive oxygen species but also by inflammation.

#### 7.4.1. PUFA and Oxidative Stress

The retina is exposed to radiant energy (e.g., light) and high oxygen consumption, leading to oxidative stress in the eye. PUFA, containing conjugated double bonds, are particularly sensitive to oxidation. Indeed, singlet oxygen can induce the peroxidation of these lipids, leading to toxic products for the eye, such as 4-hydroxynonenal (the peroxidation product of linoleic acid and arachidonic acid) [174] or carboxyethylpyrrole [175]. They can then form advanced lipid peroxidation end-products found in drusen [150].

Excessive amounts of lipofuscin are also characteristic of oxidative stress in the eye. Indeed, lysosomes maintain a daily recycling process of PUFA in photoreceptor outer segments by the RPE. Due to the high oxidative environment in the retina, lysosomes are less effective, leading to incomplete phagocytosis and the degradation of PUFA. This incomplete digestion causes the accumulation of lipofuscin and its autofluorescent fluorophore in the RPE, leading to further oxidative stress to the eye in a continuous vicious cycle [176,177].

#### 7.4.2. PUFA and Inflammation

Phospholipase A2 (PLA_2_) is an enzyme activated by ischemia, light exposure, oxidative stress, apoptosis, inflammation and aging. It hydrolyzes fatty acids located at the sn-2 position of retinal phospholipids to yield free LC-PUFA. After being activated by the same stimuli as PLA_2_, cyclooxygenases (COX) and lipoxygenases (LOX) then catalyze the conversion of free LC-PUFA to eicosanoids [152]. Eicosanoids are oxidized derivatives of 20-carbon PUFA [178], which include, among others, prostaglandins (PG), leukotrienes (LT) and thromboxanes (TX). They mainly act locally and modulate vascular functions of cells (choroidal vessels in the case of the retina) and inflammation [178].

Both omega-3 and omega-6 fatty acids have an impact on eicosanoid metabolism, resulting in opposite effects: omega-6 fatty acids are the source of predominantly pro-inflammatory metabolites and omega-3 are the source of anti-inflammatory metabolites (Figure 4). These fatty acids interact with COX (production of PG, TX and resolvins) and LOX (production of LT, resolvins and protectins) [179].

AA is the source of pro-inflammatory mediators metabolized by COX-1 and COX-2, such as PGE_2_ and TXA_2_, that increase vascular permeability [164,180]. These eicosanoids of series-2 are the major products, compared to series-1 and -3, because AA is the preferential substrate for COX [181].

EPA is a precursor of the anti-inflammatory LT (series-5), TX and PG (series-3) [152]. DHA is also known to generate anti-inflammatory lipid mediators of the docosanoid family, such as D-series resolvin (D1, D4) and neuroprotectin D1 [156]. In addition, EPA and DHA decrease or prevent the increase in InterCellular Adhesion Molecule (ICAM-1), IL-6, IL-1β and VEGF, the latter having an important role in CNV in wet AMD [182,183]. In general, EPA appears to be more effective in regulating the anti-inflammatory cytokine balance, while DHA appears to preferentially inhibit pro-inflammatory mediators [184].

Omega-3 VLC-PUFA are precursors of anti-inflammatory mediators, such as elovanoids, necessary for photoreceptor cell integrity [185].

AMD patients have a higher plasma omega-6/omega-3 ratio than healthy patients [186], which could correlate with a lack of omega-3 fatty acids. This imbalance amplifies the inflammatory mechanisms responsible for drusen formation in dry AMD. In wet AMD, an inflammatory environment promotes the formation of CNV [182].

As shown in Figure 4, the same elongation enzymes are required to produce omega-3 and omega-6 LC-PUFA but also omega-9 (oleic acid) and trans fatty acids. Thus, there is competition between these different classes of fatty acids to be processed. The preferential substrate of the elongation enzymes is EPA, an omega-3 present in very small quantities in the eye, followed by AA, an omega-6 present in much larger quantities during inflammatory phenomena or ocular pathologies such as AMD. Moreover, the eicosanoid derivatives of EPA are less biologically active than the AA derivatives [187]. It is, therefore, even more important to supply the organism with adequate amounts of foods rich in omega-3 fatty acids. Thus, upon regular consumption of DHA and EPA, series-2 and -4 eicosanoids (pro-inflammatory and pro-angiogenic) are reduced, whereas series-5 LT and series-3 PG and TX (anti-inflammatory) are increased; the omega-6/omega-3 balance decreases, shifting from a pro-inflammatory to an anti-inflammatory environment [188].

Lipid peroxidation products are a main source of ROS that increase oxidative stress but also retinal inflammation. Indeed, they can modulate and activate inflammatory cytokines. A2E, when cleaved during lipid peroxidation processes, can activate complement C2 and C3, thus increasing inflammation [189]. All these lipid products, as well as immunoglobulins and proteins involved in complement and immune response, are found in drusen, characteristic of retinal inflammation [176].

In conclusion, LC-PUFA metabolites can regulate inflammation with pro- or anti-inflammatory (omega-6 or omega-3, respectively) metabolites, but lipid peroxide products, due to oxidative stress, accentuate inflammation and retinal degeneration.

### 7.5. Interest of Omega-3 LC-PUFA and VLC-PUFA in the Prevention and the Treatment of Dry and Wet AMD: Clinical Studies

AMD is characterized by a destruction of the macula and its photoreceptors (composed of many fatty acids), but PUFA, mainly DHA, are less present in the macula than in the periphery. In addition, AMD is characterized by non-eliminated lipidic deposits originating from fatty acids of the photoreceptors, responsible for oxidative stress and local inflammation. Despite these contradictory facts, many studies reported a positive effect of the intake of fatty acids, mainly DHA and EPA, in the prevention and treatment of AMD.

Fan and Song have sought to understand more precisely the positive impact of omega-3 fatty acids in AMD and highlighted four major activities [163]. Firstly, structural replacements are determined to be ongoing, more specifically in phospholipid membranes, where omega-6 is substituted for omega-3 fatty acids. Secondly, PUFA, via the nuclear factor (erythroid-derived 2)-like 2 (NFE2L2), induces cellular resistance to oxidative stress. The third mode of action is the inhibition of choroidal neovascularization. Indeed, by activating the adiponectin pathway and inhibiting the production of matrix metalloproteinases, omega-3 fatty acids prevent pathological angiogenesis. Finally, omega-3-enriched triglyceride-rich lipoproteins reduce ROS production and RPE destruction compared to SFA-enriched triglyceride-rich lipoproteins. Thus, in the absence of omega-3 PUFA, all these mechanisms could be compromised and lead to or amplify AMD. Still, it is not yet clear whether PUFA deficiency or photoreceptor degeneration is the first to appear [167].

Different trials and meta-analyses [190,191,192] studied the impact of fatty fish, which is rich in PUFA, in the early [193,194], intermediate [188] or late [182,195,196,197] stages of pathology. A meta-analysis of nine studies concluded that an intake of omega-3 PUFA is associated with a 38% reduction in the risk of late AMD [191]. Only the AREDS2 study [198] questioned the protective effects of omega-3, whereas the AREDS study suggested a favorable association between the intake of omega-3 fatty acids and a decreased risk of AMD. This may be explained by the different patient inclusion criteria of the studies, the quantities of DHA and EPA administered and the limits of detection of the benefits [180,199].

For other fatty acids, their roles are less clear. According to some authors, omega-3 fatty acids are no longer protective when the intake of linoleic acid or trans-PUFA is high [193,200,201], while Wang et al. found that linoleic acid is the only omega-6 that would not have a negative impact on AMD [202]. Unfavorable associations between AMD and long-chain SFA would be dependent on the population and their initial dietary habits: for the Japanese, who consume mainly fatty fish, increased SFA intake is correlated to a reduced risk of developing early AMD [203]. In other countries, high consumption of long-chain SFA is correlated to a higher risk of developing AMD, but not in a statistically significant way for the majority of studies [143]. Some authors have found a contradictory impact of MUFA depending on the stage of AMD [204,205]. Medium-chain SFA would not have a negative impact [206]. Table 2 lists the main findings of clinical trials evaluating the impact of fatty acids on AMD.

Thanks to the results of all these clinical trials, it is now accepted that omega-3 LC-PUFA has a positive impact on visual function. Further studies need to be carried out on omega-6, such as linoleic acid or MUFA, due to opposing research results.

There are current recommendations for PUFA intake with the objective of maintaining good visual acuity: consumption of fatty fish at least twice a week is recommended. Even if detection at the retinal level is difficult, the use of biomarkers with high predictive performance for retinal content [210] and numerous clinical trials have led European authorities to recommend a daily oral dose of 250 mg of DHA. Many food supplements on the market contain a mixture of DHA and EPA in different ratios [211]. Benefits still occur for omega-3 PUFA concentrations between 240 and 350 mg/day in other studies [6]. The most recent dietary supplements contain, in addition to omega-3 fatty acids, vitamins (C, E), minerals (zinc), lutein, zeaxanthin and resveratrol, having also demonstrated a positive impact on the pathology [212].

## 8. Conclusions

AMD being a complex pathology, the exact initial mechanisms remain the subject of debate. Indeed, under the effect of environmental factors (smoking), internal factors (age, genetics) and various stresses (oxidative stress, inflammation), retinal cells are confronted with a harmful environment. Physiological ocular aging can then switch to pathological degradation and lead to dry or wet AMD. Moreover, with the world’s aging population, an increase in the incidence of AMD could occur in the coming years.

Fatty acids, a main component of photoreceptors, are significantly reduced in both types of AMD. Unfortunately, it is still not clear which mechanism—photoreceptor degeneration or fatty acid deficiency—appears first. In any case, despite the apparent contradictions between the known mechanisms of AMD (accumulation of lipid deposits at the RPE destroying the RPE cells and photoreceptors) and the additional intake of lipids in the retina, an efficiency has been demonstrated in the pathology when supplementing with omega-3 LC-PUFA and VLC-PUFA. Indeed, thanks to their role in the composition of photoreceptors as well as their antioxidant, anti-proliferative but also anti-angiogenic properties, omega-3 fatty acids can limit the degeneration of the retina. This supplementation would be even more interesting during the early stages or for prevention in the presence of known risk factors.

## 9. Future Directions and Challenges

Current treatments concern mainly wet AMD, with the administration of anti-VEGF by intravitreal injections. Extremely effective but very invasive, intravitreal injections can lead to serious side effects. Still, it is important to only administer these anti-VEGF drugs locally and not orally or systemically because the inhibition of VEGF in other areas of the body could lead to poor development of vessels essential for proper organ function. For dry AMD, mostly food supplements containing several minerals, vitamins and omega-3 fatty acids are recommended and used in Europe. The drawback is that, with oral administration, the bioavailability of lipophilic molecules such as fatty acids is extremely low [213,214,215], considerably reducing the therapeutic effectiveness in the eye.

LC-PUFA and VLC-PUFA are an important therapeutic approach for the treatment of both types of AMD, and it would be interesting to develop new galenic forms with these active ingredients. As the intravitreal route is resented by patients and the oral route displays limited bioavailability, it could be interesting to develop an ocular form for topical application. This outpatient treatment has numerous advantages, such as ease of administration, easy adaptation of the dosage, adaptation to the patient’s lifestyle and better compliance. The topical route would, therefore, counteract the causes of non-adherence to intravitreal injection treatments.

Physiologically, reaching the posterior segment of the eye topically is indeed challenging due to the presence of numerous ocular barriers (corneal barrier, blood–retinal barrier, blood–aqueous barrier) and the eye’s defense mechanisms (palpebral blinking, rapid renewal of the tear film, aqueous humor, vitreous humor). There is then a need to develop an ophthalmic form that considers all of these parameters and allows for a prolonged residence time in the ocular zones of interest. With this objective, nanotechnologies, hydrogels or modified release forms can be developed. Among these galenic forms, many allow the simultaneous encapsulation of lipophilic and hydrophilic molecules. Numerous possibilities in the choice of raw materials or combinations are then offered: an association of fatty acids and anti-VEGF for the wet form, a combination of fatty acids, minerals and vitamins for the dry form or a combination of fatty acids and all relevant molecules in both forms of AMD.

Various authors have been interested in the development of nanotechnological ophthalmic forms for topical application to target diseases in the posterior segment of the eye, but few articles specifically target AMD with adapted active ingredients and retinal cell reaching. Further research on this specific topic of treating AMD with fatty acids for a topical ocular administration could make it possible to develop an effective treatment for patients with the objective of permanently treating AMD.

## Figures and Tables

**Table 1:** Current commercialized treatments (in Europe) and in-development molecules (worldwide) for dry and wet AMD.

Treatment			Administration Mode	Mechanism of Action	Refs.
Wet AMD					
Commercialized (Europe)	Laser photocoagulation		Local laser	Destruction of extrafoveolar neovessels under effect of temperature (thermal argon laser).	[59,72]
	Verteporfin (Visudyne^®^) + photodynamic therapy		Oral administration + local laser	Destruction of neovessels by applying local infrared radiation to a photosensitive molecule.	[59]
	Anti-VEGF	Ranibizumab (Lucentis^®^), Bevacizumab (Avastin^®^) ^1^, Aflibercept (Eylea^®^), Brolucizumab (Beovu^®^), Faricimab (Vabysmo^®^)	Intravitreal injection	Inhibition of VEGF (mainly VEGF-A) by binding to them and blocking their interactions with receptors.	[73,74,75,76]
In development (worldwide)	Anti-VEGF	KSI-301 (phase III done, waiting for results)	Intravitreal injection	Inhibition of VEGF (mainly VEGF-A).	[77,78]
	Tyrosine kinase inhibitors	Vorolanib (Eyp-1901, phase II ongoing)	Intravitreal injection	Inhibition of VEGFR tyrosine kinase.	[79]
		Axitinib (CLS-AX, phase II ongoing)	Suprachoroidal injection		[80]
		Axitinib (OTX-TKI, phase III ongoing)	Ocular implant		[81]
	Anti-complements	Iptacopan (phase II ongoing)	Oral administration	Inhibition of C5 or complement factor B.	[82]
	Anti-VEGF and anti-complement	IBI302 (phase II ongoing)	Intravitreal injections	Bispecific fusion protein acting as a decoy: inhibition of VEGF-mediated signaling pathway and complement factors C3b and C4b.	[83]
	Gene therapy	RGX-314 (phase II ongoing)	Subretinal injection	Adeno-associated virus (AAV) vector delivering a gene encoding for anti-VEGF Fab portion.	[84]
		ADVM-022 (phase II ongoing)	Intravitreal injection	AAV vector encoding aflibercept.	[85]
		RetinoStat^®^ (phase I ongoing)	Subretinal injection	Lentivirus delivering anti-angiogenic genes (endostatins and angiostatin).	[86]
Dry AMD					
Commercialized (Europe)	Food supplements	Macular pigments, vitamins, minerals, antioxidants, fatty acids	Oral administration	Supplementation of minerals and fatty acids necessary for the development and function of photoreceptors.	[87,88]
In evaluation by the European Medicines Agency	Anti-complements	Pegcetacoplan (Syfovre^®^) ^2^	Intravitreal injection	Inhibition of C3 complement factors.	[89]
		Avacincaptad pegol (Izervay^®^)	Intravitreal injection	Inhibition of C5 complement factors.	[90]
In development (worldwide)	Vitamin supplements	ALK-001 (phase III ongoing)	Oral administration	Replacement of natural vitamin A by chemically modified vitamin A to prevent formation of vitamin A dimers.	[91]
	Anti-TNFα	ONL-1204 (phase I ongoing)	Intravitreal injection	Inhibition of Fas receptor (TNFalpha family).	[92]
	Anti-complements	IONIS-FB-LRx (phase II ongoing)	Subcutaneous administration	Inhibition of complement factor B.	[93]
		Danicopan (ALXN2040, phase II ongoing)	Oral administration	Inhibition of complement factor D.	[94]
	Gene therapy	GT005 (phase II ongoing)	Subretinal injection	AAV vector encoding for complement factor I proteins.	[95]
	Cellular therapy	OpRegen (phase I/IIa ongoing)	Subretinal surgery	Administration of human embryonic stem cell to regenerate retinal pigment epithelial.	[96]
	Retinal implant + medical device (glasses): Prima technology		Subretinal implant	Implantation of a photovoltaic matrix which simulates photoreceptors.	[97,98,99]

^1^ Avastin^®^ is used off-label in Europe. ^2^ The European Medicines Agency (EMA) refused the marketing authorization of Syfovre in January 2024 because the risks were higher than the benefits. The company requested a reevaluation of its opinion.

**Table 2:** Clinical trials evaluating the impact of fatty acids on AMD.

Study	Objective	Stage of AMD	Number of Participants	Conclusion	References
Blue Mountain Eye Study	To assess the relationship between baseline dietary fatty acids and 10-year incident AMD.	Early	2454	Protective effect against early AMD with regular fish consumption, high consumption of omega-3 PUFA and low intake of foods rich in linoleic acid.	[194]
Polanut study	To assess the associations of dietary fat with the risk of ARM.	Early	832	Increased risk of ARM with high total, saturated and monounsaturated fat intake. Reduced risk (60%) of ARM with fatty fish intake (more than once a month).	[205]
Alienor study	To report the associations of ARM with past dietary intake in elderly French subjects.	Early	1289	Decreased risk of ARM in subjects with high intake of omega-3 LC-PUFA.	[207]
Melbourne Collaborative Cohort Study	To evaluate the association between fat intake and prevalence of AMD.	Early and late	6734	Decreased risk of AMD with a diet low in trans-unsaturated fat and rich in omega-3 fatty acids and olive oil.	[193]
Nurses’ health study and the health professional follow-up study	To evaluate the association between intake of EPA and DHA and the intermediate and advanced stages of AMD.	Intermediate and late	114,850	Prevention or delay of visually significant intermediate AMD with higher intake of EPA. No association with advanced AMD.	[188]
US twin study of AMD	To evaluate modifiable risk and protective factors for AMD among elderly twins.	Intermediate and late	681	Increased risk of AMD with cigarette smoking. Reduced risk of AMD with fish consumption and omega-3 fatty acid intake.	[200]
NAT2 study	To evaluate the efficacy of DHA-enriched oral supplementation in preventing exudative AMD.	Late	263	Reduction of CNV incidence with high EPA plus DHA index.	[208]
Eye-risk consortium	To investigate association of adherence to the Mediterranean diet with incidence of advanced AMD.	Late	4996	Risk reduction (41%) of incidence of advanced AMD with higher adherence to the Mediterranean diet.	[209]
European Eye study	To examine association between adherence to a Mediterranean diet and prevalence of AMD in countries ranging from Southern to Northern Europe.	Late	5060	Protective effect of adherence to a Mediterranean diet in late AMD patients.	[196]
AREDS	To investigate whether omega-3 LC-PUFA intake was associated with a reduced likelihood of developing central GA and neovascular AMD.	Late	1837	Lower 12-y incidence of central GA and neovascular AMD in participants at moderate-to-high risk for those reporting the highest consumption of omega-3 LC-PUFA.	[197]
AREDS 2	To determine whether adding lutein + zeaxanthin, DHA + EPA or both to the AREDS formulation decreases the risk of developing advanced AMD and to evaluate the effect of eliminating beta carotene, lowering zinc doses or both in the AREDS formulation.	Late	4203	No risk reduction of advanced AMD with addition of lutein + zeaxanthin, DHA + EPA or both to the AREDS formulation.	[198]

## Abbreviations

AA, arachidonic acid; AAV, adeno-associated virus; ALA, α-linolenic acid; AMD, Age-related Macular Degeneration; ApoE, apolipoprotein E; ARM, age-related maculopathy; CFH, complement factor H; CNV, choroidal neovascularization; COX, cyclooxygenase; DHA, docosahexaenoic acid; EMA, European Medicines Agency; EPA, eicosapentaenoic acid; GA, geographic atrophy; HDL, high-density lipoprotein; HUVEC, human umbilical vascular endothelial cells; iPSC, induced pluripotent stem cell; LA, linoleic acid; LC-FA, long-chain fatty acids; LC-PUFA, long-chain polyunsaturated fatty acids; LOX, lipoxygenase; LT, leukotriene; MUFA, monounsaturated fatty acids; NADPH, nicotinamide adenine dinucleotide phosphate; PG, prostaglandin; PLA_2_, phospholipase A2; PUFA, polyunsaturated fatty acids; ROS, reactive oxygen species; RPE, retinal pigment epithelium; SFA, saturated fatty acids; TLR, toll-like receptor; VLC-FA, very long-chain fatty acids; VLC-PUFA, very long-chain polyunsaturated fatty acids; TX, thromboxane; VEGF, vascular endothelial growth factor.
